# Blood lead is significantly associated with metabolic syndrome in Korean adults: an analysis based on the Korea National Health and Nutrition Examination Survey (KNHANES), 2008

**DOI:** 10.1186/1475-2840-12-9

**Published:** 2013-01-09

**Authors:** Sang Youl Rhee, You-Cheol Hwang, Jeong-taek Woo, Dong Hyun Sinn, Sang Ouk Chin, Suk Chon, Young Seol Kim

**Affiliations:** 1 Department of Endocrinology and Metabolism, Kyung Hee University School of Medicine, 1 Hoegi-dong, Dongdaemoon-gu, Seoul, 130-702, South Korea; 2 Department of Medicine, Inje University College of Medicine, Seoul, South Korea

**Keywords:** Metabolic syndrome X, Lead, Metals, Heavy, Insulin resistance, Korea

## Abstract

**Background:**

Although an association between low-level environmental heavy metal exposure and the incidence of metabolic syndrome (MS) has been hypothesized, little research on this topic has been conducted on a population-wide level.

**Methods:**

We analyzed MS status and whole blood lead, mercury, cadmium, manganese, and creatinine-adjusted urine arsenic concentrations in 1,405 subjects, ≥ 20 years of age, who were registered for the Korea National Health and Nutrition Examination Survey, 2008.

**Results:**

Various demographic and biochemical parameters were associated with MS and blood heavy metal status. After adjusting for these variables, lead was the only heavy metal that was significantly associated with MS. Lead concentrations in subjects with MS were significantly higher than those in subjects without MS (*p* = 0.015). The prevalence of MS and a moderate/high risk for cardiovascular disease, as determined by Framingham risk score, also increased significantly according to the logarithmic transformation of the lead quartile (*p* < 0.001). The odds ratios and 95% confidence intervals for MS were 1.56 (0.90–2.71), 1.63 (0.94–2.83), and 2.57 (1.46–4.51) for the second, third, and fourth quartiles of the log-transformed lead quartile, respectively, as compared with those of the lowest quartile after multiple adjustments for confounding factors. Serum triglyceride level was the only MS diagnostic component significantly associated with lead level in a multiple linear regression analysis (*p* = 0.006).

**Conclusions:**

These findings suggest that a higher prevalence of MS is associated with higher blood lead levels in the Korean population.

## Introduction

Tremendous changes in lifestyles and environments have occurred through rapid industrialization, and these changes have significantly affected human health. Morbidity and mortality caused by nutritional deficiencies and contagious diseases have decreased sharply, whereas obesity, diabetes mellitus (DM), and malignant tumors have become highly prevalent [1,2]. Various factors relevant to these chronic diseases have been identified as risk factors after a great deal of research [3,4]. In particular, various environmental factors other than conventional risk factors have been proposed to be independently related to both the onset and progression of chronic diseases. Moreover, it is believed that various environmental factors have an additive or synergistic effect on chronic diseases in combination with conventional risk factors [5,6].

Some studies have suggested that environmental exposure to heavy metals, such as lead and arsenic, is closely associated with the prevalence of chronic diseases, such as hypertension, DM, and cardiovascular disease (CVD) [7-9]. However, these studies focused on a limited number of subjects from a few areas; the association between environmental exposure to heavy metals and chronic metabolic diseases has rarely been investigated using a population-based approach. Consequently, little is known about how environmental exposure to heavy metals affects various chronic diseases.

Raw data from the Korea National Health and Nutrition Examination Survey (KNHANES) IV, which was conducted by the Korea Centers for Disease Control and Prevention, were released in 2011. The KNHANES IV data include blood and urine assays for heavy metals. Our aim in this study was to determine if there was an association between environmental exposure to heavy metals and metabolic syndrome (MS). Our analyses were based on the KNHANES 2008 data, which were collected from a representative sample of the noninstitutionalized civilian population in Korea.

## Methods

### Study subjects

KNHANES is a nationwide, population-based, and cross-sectionally designed health survey conducted by the Korea Centers for Disease Control and Prevention. After the first KNHANES was conducted in 1998, the second, third, and fourth surveys were conducted in 2001, 2005, and 2007–2009, respectively.

We utilized the KNHANES 2008 data, which included information on MS diagnoses and heavy metal sampling. Heavy metal sampling was conducted on 2,000 subjects by randomly selecting 10 subjects from each survey unit according to age and sex. Blood samples were collected for analyses of lead, mercury, cadmium, and manganese concentrations, and urine samples were collected to assess total arsenic concentrations.

Subjects whose age ranged from ≥ 20 years to < 80 years were selected from among the group of subjects who were tested for heavy metals (n = 1,791). Within this group, people who took medications for hypertension, DM, or dyslipidemia (*n* = 307) were excluded to eliminate the effects of medications on MS. We also excluded patients with active chronic diseases that might have contributed to MS (*n* = 51). In addition, we excluded subjects with demographic and clinical variables missing from their health interview and health examination (*n* = 28) records, including body mass index (BMI), education, occupation, physical activity, and other variables for MS diagnosis and CVD risk estimation. We included patients with cerebrovascular disease, CVD, active tuberculosis, chronic obstructive pulmonary disease, chronic kidney disease, chronic liver disease, and malignancy. Thus, data from 1,405 people were analyzed.

### Study methods

The subjects were subdivided according to the characteristics of each variable to determine differences based on demographic and clinical characteristics. The Modified National Cholesterol Education Program Adult Treatment Panel III criteria were used for subjects with a diagnosis of MS [10]. However, waist circumference measurements of ≥ 90 cm in males and ≥ 85 cm in females were selected in the MS diagnosis based on the criteria of the Korean Society for the Study of Obesity [11]. The 10-year CVD risk of subjects was also assessed using the Framingham risk score [12]. Based on these results, subjects were classified into low-risk (< 5%), moderate-risk (5% to < 10%), and high-risk groups (≥10%) [10]. Current smokers were defined as those who had smoked more than five packs of cigarettes during their lifetime and were smoking at the time of the survey; all others subjects were defined as non-smokers. Regular alcohol drinkers were those who currently drank alcohol more than once per month, and all others were defined as non-drinkers. The 16 residential areas of the KNHANES were classified into two groups: 1) urban areas, including metropolitan cities such as Seoul, Busan, Daegu, Incheon, Gwangju, Daejeon, and Ulsan, as well as metropolitan areas such as Gyeonggi province; and 2) rural areas, comprising Gangwon, Chungbuk, Chungnam, Jeonnam, Jeonbuk, Gyeongbuk, Gyeongnam, and Jeju provinces. Educational status was categorized as follows: 1) graduation from elementary school or lower; 2) graduation from middle school; 3) graduation from high school; and 4) graduation from college or higher. Seven occupation types were recognized: 1) managers, professionals, technicians, and associated professionals; 2) clerical support workers; 3) service and sales workers; 4) skilled agricultural, forestry, and fishery workers; 5) craft and related trades workers, plant and machine operators, and assemblers; 6) elementary occupations; and 7) housewife, student, or unemployed, based on the 6th Korean Standard Classification of Occupations from the Korean National Statistical Office, which was created by following the International Standard Classification of Occupations of the International Labor Organization [13]. Physical activity of the subjects was investigated by evaluating their participation in recreational physical activity during the 1 week prior to the survey and categorized as follows: 1) none, no or minimal activity; 2) mild, > 30 minutes of walking more than 5 days per week; 3) moderate, > 30 minutes of physical activity in which the subject was tired compared to normal or breathing slightly hard more than 5 days per week; and 4) vigorous, > 20 min of vigorous physical activity in which the subject was exhausted compared to normal or breathing hard more than 3 days per week.

Samples from all subjects were collected after a period of fasting for 8 hours or longer. Specimens were immediately transported to the central laboratory (NeoDIN Medical Institute, Seoul, Korea), where they were analyzed within 24 hours. This central laboratory is certified by the Ministry of Labor of Korea as one of the designated laboratories for assaying the levels of special chemicals, including heavy metals. For external quality assurance and control, this laboratory is required to fulfill all the requirements of the Quality Assurance Program operated by the Korea Occupational Safety and Health Agency, and to have passed the German External Quality Assessment Scheme.

Biochemical measurements, including total cholesterol, triglycerides, high-density lipoprotein (HDL) cholesterol, blood urea nitrogen (BUN), creatinine, aspartate aminotransferase (AST), alanine aminotransferase (ALT), and fasting plasma glucose concentrations were assessed using an automated analyzer (Hitachi Automatic Analyzer 7600; Tokyo, Japan) with enzymatic assays. HDL cholesterol was evaluated using standard samples as equivalents between the KNHANES central laboratory and the U.S. Centers For Disease Control and Prevention to produce an accurate lipid profile. The differences between the two laboratories were adjusted for by Passing–Bablok regression [14]. Low-density lipoprotein (LDL) cholesterol levels were calculated using the Friedewald equation [15]. Serum insulin concentrations were measured using a gamma counter (1470 Wizard, Perkin Elmer, Turku, Finland) and an immunoradiometric assay (Biosource, Nivelles, Belgium).

Trace element EDTA tubes (BD, Franklin Lakes, NJ, USA) were used to store blood samples for the heavy metal assays. More than 20 mL of clean midstream urine was collected as the urine sample. Lead, cadmium, manganese, and urine total arsenic concentrations were measured with a graphite furnace atomic absorption spectrometer (Analyst 600, Perkin Elmer). The limits of detection were 0.223 μg/L for lead, 0.087 μg/L for cadmium, 0.16 μg/L for manganese, and 1.679 μg/L for arsenic. The concentrations in all samples were higher than the limits of detection. The inter-assay coefficients of variation were 2.2–6.0% for lead, 3.0–11.9% for cadmium, 2.2–4.8% for manganese, and 2.5–3.2% for the urine arsenic assay. Urine creatinine-adjusted arsenic values were calculated to correct for the effect of differences in creatinine clearance among the subjects. Blood mercury concentrations were measured using the gold amalgam method (DMA-80, Milestone, Sorisole, Italy). The limit of detection for mercury was 0.05 μg/L, and the concentrations of all samples were higher than the limit of detection. The inter-assay coefficient of variation for the mercury assay was 1.1–4.1%.

### Statistical analysis

Differences in blood and urine heavy metal concentrations based on MS status were investigated based on the health interview and health examination data from KNHANES 2008. The difference in the prevalence of MS and high CVD risk was examined by subdividing the subjects into quartiles based on heavy metal concentrations. Odds ratios (ORs) and 95% confidence intervals (CIs) for MS were estimated according to the quartile for each heavy metal concentration. Finally, the correlations between each MS diagnostic component and blood heavy metal concentrations were evaluated.

PASW (version 18.0) (SPSS, Inc., Chicago, IL, USA) was used for statistical analyses and data management. Demographic and biochemical data are presented as either means ± standard deviations (SD) or proportions, while heavy metal concentrations are presented as geometric means ± 95% confidence intervals (CI) [16]. An independent sample *t*-test and chi-square test were used to determine statistical significance with respect to MS status. A linear-by-linear association test was conducted to verify the significance of the trends for the prevalence of MS and moderate (5% to < 10%) and high (≥ 10%) CVD risk subgroups with respect to the quartile for each heavy metal concentration.

Differences in blood and urine heavy metal concentrations based on MS status were determined, and the influence of other variables was adjusted for by using analysis of covariance. ORs for the prevalence of MS were calculated using the lowest quartile subgroup as the standard, and the effect of confounders was adjusted for by using logistic regression to investigate the degree of MS risk based on heavy metal concentration. Linear trends for ORs were obtained from the significance of the continuous version of these quartiles entered into the same model. Multiple linear regression models were used to clarify the relationships between the diagnostic components of MS and each heavy metal concentration. *P*-values < 0.05 were considered statistically significant.

### Ethics statement

Because this study analyzed publicly available data sets, it exempted from institutional review board approval.

## Results

### Demographic and clinical characteristics of subjects according to MS status

A significant difference in demographic and clinical characteristics was observed among subjects stratified according to MS status (Additional file 1: Table S1). The mean age (*p* < 0.001) and BMI (*p* < 0.001) of MS subjects were significantly higher than those of non-MS subjects. Furthermore, the proportions of males (*p* < 0.001), current smokers (*p* = 0.010), and subjects with low educational status (*p* < 0.001) were significantly higher in the group of MS subjects than the group of non-MS subjects. In addition to these variables, the proportion of subjects with a moderate/high CVD risk according to the Framingham risk score (*p* < 0.001) was significantly higher among MS subjects than subjects without MS. Considerable differences among the subjects were observed for the other biochemical parameters, including total cholesterol (*p* < 0.001), creatinine (*p* = 0.008), AST (*p* < 0.001), ALT (*p* < 0.001), and fasting insulin (*p* < 0.001), which were not included as MS diagnostic criteria. In contrast, alcohol, location, occupation, and physical activity, as well as BUN and LDL cholesterol levels, were not significantly different between MS and non-MS subjects.

### Differences in blood and urine heavy metal concentrations by MS status

Heavy metal concentrations in subjects were compared based on MS status. The results of the independent sample *t*-test showed that lead, mercury, cadmium, and creatinine-adjusted urine total arsenic concentrations were significantly higher in subjects with MS (data not shown) than in those without MS. However, a significant difference was found only for the lead concentration after other clinical and biochemical parameters affecting MS status were adjusted for in the analysis (*p* = 0.015, Table 1). The concentration of mercury tended to be higher in subjects with MS than those without MS, but the difference was not significant (*p* = 0.082).

**Table 1:** Blood and urine heavy metal concentrations in study subjects according to metabolic syndrome (MS) status adjusted for all other significant variables

	**MS (−)**	**MS (+)**	***p***
	**n = 1,170**	**n = 235**	
Lead (μg/dL)	2.28 (2.23-2.33)	2.45 (2.33-2.58)	0.015
Mercury (μg/dL)	4.62 (4.49-4.76)	4.96 (4.62-5.32)	0.082
Cadmium (μg/L)	0.85 (0.83-0.88)	0.88 (0.82-0.95)	0.401
Manganese (μg/dL)	1.29 (1.27-1.31)	1.33 (1.28-1.38)	0.126
Arsenic (μg/g creatinine)	111.2 (106.9-115.6)	115.5 (105.3-126.6)	0.464

Expressed as geometric mean (95% confidence intervals).

Adjusted for age, sex, smoking, education, total cholesterol, creatinine, aspartate amino transferase, alanine aminotransferase, and fasting serum insulin by analysis of covariance.

### Prevalence of MS in moderate/high CVD risk subjects according to lead concentration

**Figure 1:**
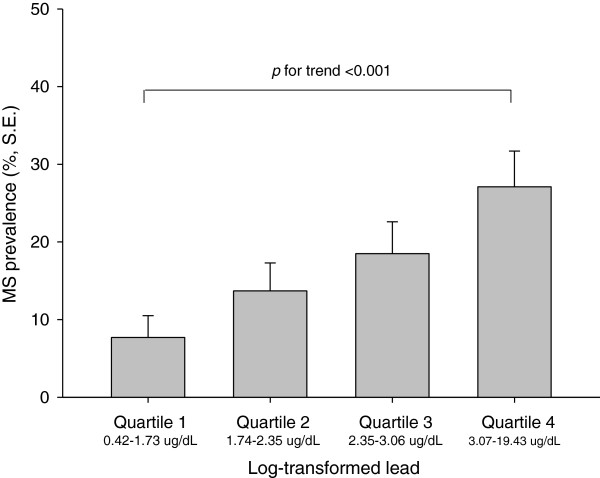
Prevalence of metabolic syndrome by log transformed lead concentration quartile.

**Figure 2:**
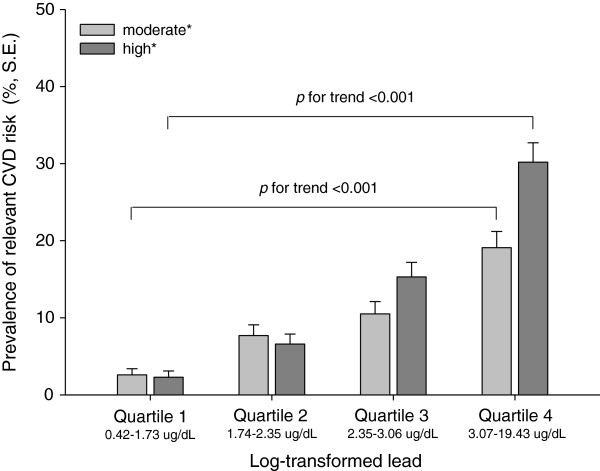
**Prevalence of moderate (5 to < 10%) and high ( **≥ **10%) 10-year cardiovascular disease risk subjects by log transformed lead concentration quartile.**

The prevalence of MS in subjects according to the log-transformed lead quartiles increased from 7.7% to 13.7%, 18.5%, and 27.1%, respectively (Figure 1). The proportion of moderate CVD risk subjects, calculated based on the Framingham risk score, increased from 2.6% to 7.7%, 10.5%, and 19.1% based on the lead quartiles. High CVD risk subjects showed increases from 2.3% to 6.6%, 15.3%, and 30.2% based on lead quartiles (Figure 2). The prevalence of MS and the proportion of moderate/high CVD risk subjects showed trends that were significant according to the lead quartile (*p* < 0.001).

### Difference in MS risk based on lead concentration

We next evaluated the ORs and 95% CIs for MS prevalence according to the log-transformed lead quartiles (Table 2). The ORs and 95% CIs for MS of the second, third, and fourth quartiles compared to the lowest quartile were 1.54 (0.93–2.56), 1.82 (1.10–3.01), and 2.50 (1.50–4.15), respectively, after adjusting for demographic characteristics, and a significant increase in the trend for the OR was observed (*p* < 0.001). After adjusting for biochemical variables, the OR and 95% CI for the highest quartile was 2.57 (1.46–4.51), which was still significant, and the trend for the numbers was also significant (*p* < 0.001). The ORs for the log-transformed mercury quartiles increased significantly after adjustment for demographic variables (*p* = 0.045, Additional file 1: Table S2). However, no significant trend was observed after additional adjustment for biochemical parameters (*p* = 0.365, Additional file 1: Table S2), and no remarkable findings were observed for the other heavy metals.

**Table 2:** Adjusted ORs of the study subjects with metabolic syndrome according to logarithmic transformed lead quartiles

	Model 1*	***p***	Model 2**	***p***
	**OR (95% CI)**		**OR (95% CI)**	
Quartile 1	Referent	-	Referent	-
Quartile 2	1.54 (0.93-2.56)	0.095	1.56 (0.90-2.71)	0.117
Quartile 3	1.82 (1.10-3.01)	0.020	1.63 (0.94-2.83)	0.081
Quartile 4	2.50 (1.50-4.15)	<0.001	2.57 (1.46-4.51)	0.001
*p* for trend***		0.001		0.001

*OR*, odds ratio; *CI*, confidence interval.

* Model 1 is shown as OR and 95% CI adjusted for age, sex, smoking, and education.

** Model 2 is shown as OR and 95% CI further adjusted for total cholesterol, creatinine, aspartate amino transferase, alanine aminotransferase, and fasting serum insulin.

*** Linear trend obtained from the significance of the continuous version of these quartiles entered into the same model.

### Correlation between the diagnostic components of MS and lead concentration

We evaluated if there was a correlation between the diagnostic components of MS and blood lead concentration (Table 3). In multivariate regression analysis, which included demographic variables, triglyceride level was significantly associated with blood lead level (*p* = 0.007). Additionally, serum triglyceride level remained significantly associated with blood lead in the multivariate regression analysis, which included demographic and biochemical confounders (*p* = 0.006). No other remarkable findings were observed for the other heavy metals (data not shown).

**Table 3:** Adjusted regression coefficients of diagnostic components for metabolic syndrome with log-transformed lead in the study subjects

	Model 1*		Model 2**	
	**Standardized β**	***p***	**Standardized β**	***p***
Abdominal circumference	0.035	0.181	0.051	0.073
Triglyceride	0.068	0.007	0.080	0.006
HDL cholesterol	0.022	0.389	0.033	0.225
Systolic BP	0.016	0.543	0.020	0.457
Fasting glucose	0.014	0.567	0.019	0.437

* Model 1, adjusted for age, sex, smoking, and education.

** Model 2, further adjusted for total cholesterol, creatinine, aspartate amino transferase, alanine aminotransferase, and fasting serum insulin.

## Discussion

We investigated the association between MS and environmental exposure to heavy metals based on the 2008 KNHANES data, which were obtained from a representative sample of the noninstitutionalized civilian population of Korea. We found that environmental factors, particularly blood lead level, as well as recognized conventional risk factors for MS significantly affected MS and its relevant pathophysiology.

Blood lead was the only heavy metal that showed a significant association with MS after adjustment for demographic and biochemical parameters. The prevalence of MS in the study subjects increased significantly according to log-transformed lead quartiles. The prevalence of moderate to high CVD risk subjects, as estimated by the Framingham risk score, also showed a significant increasing trend according to lead quartile. Moreover, the ORs of MS by lead quartile were significantly high, with trends that increased progressively according to lead quartile. Mercury, cadmium, arsenic, and other heavy metals also increased significantly in subjects with MS when the effects of confounders were not adjusted for. However, no significant differences were observed after adjustment. The other heavy metals did not demonstrate any meaningful trends in the ORs for MS when compared by quartiles. These results suggest that environmental lead exposure is closely associated with the prevalence of MS and a high CVD risk.

People can be exposed to lead through contaminated air, water, soil, food, and consumer products, and they can inhale and ingest lead [17]. After inhalation or ingestion, lead is absorbed into the body through the mucous membranes. Environmental lead is ubiquitous, which means that most people in the world probably have trace blood lead levels [18]. Lead exposure has decreased drastically since leaded gasoline was banned internationally and since the use of lead compounds began to decline in the 1970s. However, substantial amounts of lead are still utilized in industrial products [19]. About 15% of the lead to which a person is exposed is absorbed into the body; however, children, pregnant women, and patients with calcium, zinc, or iron deficiencies are likely to absorb much more [20,21]. Lead accumulates in the blood, soft tissues, and bone. Small amounts of lead are stored in the brain, spleen, kidneys, liver, and lungs [17,22]. Lead that has accumulated in the body is eliminated very slowly through the urine and is also excreted through the stool, nails, and sweat [17]. The estimated half-life of lead in bones is 20–30 years, and these organs could be a source of lead continuously released into the bloodstream [17]. The half-life of lead in the blood of adults is about 40 days. However, blood lead has a longer half-life in children and pregnant women, as their bones are rapidly remodeled, which translates into increased blood levels of lead [20]. Generally, blood lead levels are measured to evaluate lead exposure, but the total amount of accumulated lead in the body is not accurately reflected by this measurement [23]. The World Health Organization and the U.S. Centers for Disease Control and Prevention announced that blood lead levels > 10 μg/dL may affect health [20,24]. However, the definitive level for safe exposure to lead has not yet been identified, because blood lead levels < 10 μg/dL can also cause health problems [20,24].

Chronic lead exposure is detrimental to health. Chronic exposure to low-level lead can cause depletion of glutathione and protein-bound sulfhydryl groups, resulting in an increase in reactive oxygen species and damage to cell structures, including DNA and cell membranes [25]. Chronic lead exposure inhibits DNA transcription, vitamin D synthesis, and the function of enzymes that maintain cell membrane integrity. Lead also alters the permeability of blood vessels and the synthesis of collagen [26]. Additionally, lead can have harmful effects on immune system development and cause production of excessive inflammatory proteins [27]. As a result, lead may cause an increase in levels of proinflammatory mediators and lipid peroxidation, suppress nitric oxide levels, change calcium homeostasis, as well as alter autonomic function and heart rate variability, which, in turn, could increase the likelihood of hypertension and CVD [25,28].

Another remarkable finding of this study was that serum triglyceride level was the only diagnostic component of MS significantly associated with blood lead level in multiple linear regression analysis. Some researchers have found that acute or chronic lead exposure does not greatly change triglyceride levels, or may even reduce levels [29,30]. However, direct comparison of our results with investigations that focused on subjects living in areas with high endemic lead concentrations is not appropriate. A previous experimental study also reported that lead exposure causes hypertriglyceridemia, and the VA Normative Aging Study, a population-based study performed to investigate the effects of low-level lead exposure, reported similar results to our study [25,31].

Our study has some limitations. First, as KNHANES 2008 was a cross-sectional study, a causal relationship between heavy metals and MS cannot be determined. Second, the heavy metal exposure status was evaluated only by blood samples, not by bone or soft tissue samples. As a result, the measurements may not accurately reflect chronic exposure status. Third, MS is defined as a cluster of insulin resistance-associated components, such as abdominal obesity, dyslipidemia, hypertension, and hyperglycemia, and it is not clear whether MS should be regarded as a single disease entity [32]. Despite these limitations, our study results are meaningful, because they revealed a significant association between blood lead concentration and MS in a large number of subjects representative of the entire Korean population. All study subjects underwent accurate heavy metal analyses with strict quality controls, and a significant association between blood lead level and MS was identified after adjusting for demographic and social characteristics. Furthermore, because previous studies have already revealed that MS is closely related to high CVD risk, MS should be a useful clinical target for treatment, regardless of debate regarding its definition [33-35]. In fact, many clinical practice guidelines recommend MS management, and some communities and countries have been actively conducting intervention campaigns to prevent and manage MS [10,36]. Thus, our finding that low-level lead exposure is a risk factor for CVD is meaningful.

## Conclusions

We found that blood lead concentration was significantly associated with the risk for MS and CVD, and that blood lead concentration had a close relationship with the serum triglyceride level, which is one of the diagnostic components of MS. Future large-scale longitudinal studies that overcome the limitations of the current study will provide a more detailed view of the pathophysiological role of environmental exposure to lead in patients with MS.

## Abbreviations

KNHANES: Korea National Health and Nutrition Examination Survey.

## Competing interests

The authors have no competing interests to disclose.

## Authors’ contributions

All authors had access to the data and played a role in writing this manuscript: study concept and design (SYR, Y-CH); acquisition of data (SYR); analysis and interpretation of data (SYR, Y-CH, J-tW); drafting of the manuscript (SYR); critical revision of the manuscript for important intellectual content (DHS, SOC, SC, YSK); statistical analysis (SYR, Y-CH); and study supervision (J-tW). All authors read and approved the final manuscript.

## Supplementary Material

Additional file 1: Table S1 Demographic and clinical characteristics of the study subjects by metabolic syndrome. **Table S2.** Adjusted odds ratios of the study subjects with metabolic syndrome by logarithmic transformed mercury, cadmium, manganese, and arsenic quartiles. Click here for file
