# Supplementary material for: Blood lead is significantly associated with metabolic syndrome in Korean adults: an analysis based on the Korea National Health and Nutrition Examination Survey (KNHANES), 2008
Source: Cardiovasc Diabetol. 2013 Jan 9;12:9. doi: 10.1186/1475-2840-12-9 (PMC3849944; doi:10.1186/1475-2840-12-9)
Supplement: Additional file 1: Table S1 — Demographic and clinical characteristics of the study subjects by metabolic syndrome. Table S2. Adjusted odds ratios of the study subjects with metabolic syndrome by logarithmic transformed mercury, cadmium, manganese, and arsenic quartiles. [file 1475-2840-12-9-S1.docx]

**Supplement Tables**

Table S1. Demographic and clinical characteristics of the study subjects by metabolic syndrome.

|  | MS (-) | MS (+) | *p* |
| --- | --- | --- | --- |
|  | n=1,170 | n=235 |  |
| Age (yrs) | 40.3±13.7 | 47.1±13.3 | <0.001 |
| Sex (Male n, %) | 542 (46.3) | 150 (63.8) | <0.001 |
| BMI (kg/m^2^) | 22.8±3.0 | 26.2±3.1 | <0.001 |
| Smoking (n, %) |  |  |  |
| no smoker | 869 (74.3) | 155 (66.0) | 0.010 |
| current smoker | 301 (25.7) | 80 (34.0) |  |
| Alcohol (n, %) |  |  |  |
| no drinker | 474 (40.5) | 81 (34.5) | 0.093 |
| regular drinker | 696 (59.5) | 154 (65.5) |  |
| Location (n, %) |  |  |  |
| urban area | 787 (67.3) | 154 (65.5) | 0.648 |
| rural area | 383 (32.7) | 81 (34.5) |  |
| Education (n, %) |  |  |  |
| elementary school or lower | 167 (14.3) | 61 (26.0) | <0.001 |
| middle school | 108 (9.2) | 29 (12.3) |  |
| high school | 505 (43.2) | 83 (35.3) |  |
| college or higher | 390 (33.3) | 62 (26.4) |  |
| Occupation^*^ (n, %) |  |  |  |
| group 1 | 150 (12.8) | 36 (15.3) | 0.062 |
| group 2 | 101 (8.6) | 16 (6.8) |  |
| group 3 | 156 (13.3) | 34 (14.5) |  |
| group 4 | 77 (6.6) | 21 (8.9) |  |
| group 5 | 129 (11.0) | 37 (15.7) |  |
| group 6 | 113 (9.7) | 24 (10.2) |  |
| group 7 | 444 (37.9) | 67 (28.5) |  |
| Physical activity (n, %) |  |  |  |
| none | 451 (38.5) | 94 (40.0) | 0.853 |
| mild | 374 (32.0) | 69 (29.4) |  |
| moderate | 114 (9.7) | 22 (9.4) |  |
| vigorous | 231 (19.7) | 50 (21.3) |  |
| Glucose tolerance status |  |  |  |
| normal glucose tolerance | 1032 (88.2) | 96 (40.9) | <0.001 |
| impaired fasting glucose | 129 (11.0) | 112 (47.7) |  |
| diabetes mellitus | 9 (0.8) | 27 (11.5) |  |
| Hypertension |  |  |  |
| no hypertension | 1098 (93.8) | 154 (65.5) | <0.001 |
| hypertension | 72 (6.2) | 81 (34.5) |  |
| 10-year CVD risk^**^ |  |  |  |
| low (<5%) | 950 (81.2) | 124 (52.8) | <0.001 |
| moderate (5 to <10%) | 98 (8.4) | 42 (17.9) |  |
| high (≥10%) | 122 (10.4) | 69 (29.4) |  |
| Total cholesterol (mg/dL) | 183.0±32.5 | 202.3±36.0 | <0.001 |
| Triglycerides (mg/dL) | 106.6±85.6 | 239.9±171.6 | <0.001 |
| HDL cholesterol (mg/dL) | 50.4±10.6 | 40.6±7.3 | <0.001 |
| LDL cholesterol (mg/dL) | 111.3±30.5 | 113.7±40.5 | 0.373 |
| BUN (mg/dL) | 13.8±3.9 | 14.0±3.9 | 0.432 |
| creatinine (mg/dL) | 0.90±0.18 | 0.94±0.18 | 0.008 |
| AST (IU/L) | 20.8±16.6 | 26.7±20.8 | <0.001 |
| ALT (IU/L) | 19.6±15.0 | 31.6±22.9 | <0.001 |
| Fasting plasma glucose (mg/dL) | 91.1±9.9 | 107.2±26.0 | <0.001 |
| Fasting serum insulin (µIU/mL) | 8.95±3.88 | 11.93±6.99 | <0.001 |

Mean ± standard deviation or n (%), by independent sample *t*-test or chi-square test.

MS, metabolic syndrome; BMI, body mass index; CVD, cardiovascular disease; HDL, high-density lipoprotein; LDL, low-density lipoprotein; BUN, blood urea nitrogen; AST, aspartate transaminase; ALT, alanine transaminase.

^*^ Occupational group refers to the KSCO-6 classification. Group 1 indicates managers, professionals, technicians, and associate professionals; group 2, clerical support workers; group 3, service and sales workers; group 4, skilled agricultural, forestry, and fishery workers; group 5, craft and related trades workers, plant and machine operators, and assemblers; group 6, elementary occupations; group 7, housewife, student, and unemployed.

^**^ Estimated by Framingham risk score.

Table S2. Adjusted odds ratios of the study subjects with metabolic syndrome by logarithmic transformed mercury, cadmium, manganese, and arsenic quartiles.

|  | Model 1* | *p* | Model 2** | *p* |
| --- | --- | --- | --- | --- |
|  | OR (95% CI) |  | OR (95% CI) |  |
| Mercury*** |  |  |  |  |
| Quartile 1 | referent | - | referent | - |
| Quartile 2 | 0.86 (0.55-1.34) | 0.507 | 0.74 (0.46-1.20) | 0.225 |
| Quartile 3 | 0.86 (0.55-1.33) | 0.486 | 0.66 (0.41-1.07) | 0.089 |
| Quartile 4 | 1.48 (0.97-2.24) | 0.064 | 1.19 (0.76-1.87) | 0.451 |
| *p* for trend**** |  | 0.045 |  | 0.365 |
| Cadmium*** |  |  |  |  |
| Quartile 1 | referent | - | referent | - |
| Quartile 2 | 0.816 (0.524-1.271) | 0.369 | 0.77 (0.47-1.25) | 0.281 |
| Quartile 3 | 1.042 (0.669-1.622) | 0.856 | 1.07 (0.66-1.74) | 0.773 |
| Quartile 4 | 1.003 (0.625-1.608) | 0.991 | 1.09 (0.65-1.83) | 0.745 |
| *p* for trend**** |  | 0.705 |  | 0.447 |
| Manganese*** |  |  |  |  |
| Quartile 1 | referent | - | referent | - |
| Quartile 2 | 1.24 (0.83-1.87) | 0.301 | 1.35 (0.87-2.11) | 0.184 |
| Quartile 3 | 1.51 (1.00-2.27) | 0.049 | 1.42 (0.91-2.22) | 0.119 |
| Quartile 4 | 1.21 (0.78-1.88) | 0.400 | 1.22 (0.76-1.97) | 0.418 |
| *p* for trend**** |  | 0.237 |  | 0.360 |
| Arsenic*** |  |  |  |  |
| Quartile 1 | referent | - | referent | - |
| Quartile 2 | 1.19 (0.77-1.86) | 0.429 | 1.26 (0.77-2.05) | 0.359 |
| Quartile 3 | 1.44 (0.93-2.23) | 0.104 | 1.63 (1.00-2.63) | 0.048 |
| Quartile 4 | 1.24 (0.79-1.97) | 0.354 | 1.37 (0.82-2.26) | 0.227 |
| *p* for trend**** |  | 0.283 |  | 0.172 |

OR, odds ratio; CI, confidence interval.

* Model 1 is shown as OR and 95% CI adjusted for age, sex, smoking, and education.

** Model 2 is shown as OR and 95% CI further adjusted for total cholesterol, creatinine, aspartate amino transferase, alanine aminotransferase, and fasting serum insulin.

*** Log-transformed.

**** Linear trend obtained from the significance of the continuous version of these quartiles entered in the same model.
